# Predictors of mortality at 3 months in patients with skull base tumor resections in a low-income setting

**DOI:** 10.3389/fsurg.2024.1398829

**Published:** 2024-12-03

**Authors:** Mestet Yibeltal Shiferaw, Abat Sahlu Baleh, Abel Gizaw, Tsegazeab Laeke Teklemariam, Abenezer Tirsit Aklilu, Atalel Fentahun Awedew, Denekew Tenaw Anley, Bereket Hailu Mekuria, Ermias Fikiru Yesuf, Mengistu Ayele Yigzaw, Henok Teshome Molla, Mekides Muse Awano, Alemu Adise Mldie, Endeshaw Chekole Abebe, Nebyou Hailu, Sura Daniel, Dejen Teke Gebrewahd

**Affiliations:** ^1^ Department of Surgery, Neurosurgery Unit, Debre Tabor University, Debre Tabor, Ethiopia; ^2^ Department of Surgery, Neurosurgery Unit, Addis Ababa University, Addis Ababa, Ethiopia; ^3^ Department of Surgery, Debre Tabor University, Debre Tabor, Ethiopia; ^4^ Department of Public Health, College of Health Sciences, Debre Tabor University, Debre Tabor, Ethiopia; ^5^ Department of Surgery, Neurosurgery Unit, Debre Birhan University, Debre Birhan, Ethiopia; ^6^ Department of Surgery, Neurosurgery Unit, Hawassa University, Hawassa, Ethiopia; ^7^ Department of Surgery, Neurosurgery Unit, Jimma University, Jimma, Ethiopia; ^8^ Department of Clinical Health Science - Precision Health, University of South Australia, Adelaide, SA, Australia

**Keywords:** Ethiopia, meningioma, mortality, pituitary adenoma, resection, skull base tumors

## Abstract

**Objective:**

Globally, skull base tumors are among the most challenging tumors to treat and are known for their significant morbidity and mortality. Hence, this study aimed to identify robust associated factors that contribute to mortality of patients following surgical resection for a variety of skull base tumors at the 3-month follow-up period. This in turn helps devise an evidence-based meticulous treatment strategy and baseline input for quality improvement work.

**Methodology:**

A retrospective cohort study of patients undergoing skull base tumor resection was conducted at two large-volume neurosurgery centers in Ethiopia. The categorical variables were expressed in frequencies and percentages. Normal distribution of continuous data was checked by histogram and the Shapiro–Wilk test. Median with interquartile range (IQR) was calculated for skewed data, while mean with standard deviation (SD) was used for normally distributed data. Odds ratio and adjusted odds ratio (AOR) were used to express the result of univariate and multivariate binary logistic analyses, respectively. A *p*-value <0.005 was considered statistically significant at 95% confidence interval (CI).

**Result:**

The study involved 266 patients. Of this, women accounted for 63.5% of patients. The median age of patients was 37 (±IQR = 17) years while the median size of the tumor in this study was 4.9 (±IQR 1.5) cm. The mean duration of symptoms at time of presentation was 17.3 (±SD = 11.1) months. Meningioma, pituitary adenoma, and craniopharyngioma contributed to 68.4%, 19.2%, and 9% of the skull-based tumors, respectively. Mortality following skull base tumor resection was 21.1%. On multivariable binary logistic regression analysis, intraoperative iatrogenic vascular insult (AOR = 28.76, 95% CI: 6.12–135.08, *p* = 0.000), intraventricular hemorrhage (AOR = 6.32, 95% CI: 1.19–33.63, *p* = 0.031), hospital-associated infection (AOR = 6.96, 95% CI: 2.04–23.67, *p* = 0.002), and extubation time exceeding 24 h (AOR = 12.89, 95% CI: 4.89–40.34, *p* = 0.000) were statistically significant with 3-month mortality.

**Conclusion:**

Mortality from skull base tumor resection remains high in our setting. Holistic pre-operative surgical planning, meticulous intraoperative execution of procedures, and post-operative dedicated follow-up of patients in a neurointensive care unit alongside quality improvement works on identified risks of mortality are strongly recommended to improve patient outcomes. The urgent need for setup improvement and further training of neurosurgeons is also underscored.

## Introduction

Globally, brain tumors are among the top tumors known for their associated significant morbidity, mortality, disability, and economic burden. According to an estimate made in 2020 by the global cancer observatory, brain tumors ranked 19th and 12th leading cancer burden and cancer-related mortality, respectively (1). A similar study conducted in 2019 underscored the high case fatality rate of brain cancers despite their being relatively less common than other tumors. This study further highlighted the need of a high level of expertise and operative instruments, which further widens the gap in the multimodal care administered and the treatment outcomes between low-income and high-income settings, fostering an agenda for global neurosurgery (2–4).

In a subgroup analysis that was meant for looking at the differences in the incidence, trends, and mortality rates of brain cancers across continents, it was found that the African continent had the lowest incidence and mortality rate from brain cancers while the western pacific region had the highest. This major difference in the incidence of brain tumor cases and mortality from brain cancers in low-income settings compared with the high-income settings was mainly attributed to the delayed diagnosis, lack of adequate advanced diagnostic imaging tools, and limited response of treatment outcome when intervention was delayed (1, 5). However, the use of outcome measures like mortality-to-incidence ratio made clear that low-income settings like the African and Southeast Asian Regions had far higher mortalities from brain cancers compared with the high-income settings like the Western Pacific and European regions; further underscoring the major disparities of care provision and outcomes of brain tumors in the global neurosurgery (1, 6).

Diving deeper in to the sub-Saharan subcontinent, there is a major challenge facing the provision of neuro-oncological services due to shortage of neurosurgical workforces, radiation therapy, diagnostic imaging modalities, and gadgets for optimal intraoperative execution of the planned procedures (7). In this regard, the Ethiopian neuro-oncologic service provision ranks as one of the least in the region in particular and in the world in general. Furthermore, looking into the morbidity and mortality associated with skull base tumors remains overlooked and complex due to the need for a highly specialized technique, advanced operating instruments, and multimodal therapeutic modalities in an intricate intracranial region, the skull base (7).

Notably, there has been limited, if any, availability of comprehensive clinical investigations on the overall incidence of mortality and associated factors of mortality from skull base brain tumors’ resection. The same holds true for the Ethiopian neurosurgical setting as well. Perhaps because of the low neurosurgical work force and the young medical specialty that neurosurgery is in the country, there has never been a study conducted to look for the burden and predictors of mortality in patients that underwent resection for skull base brain neoplasms. Hence, this study is indispensable in the global neurosurgery arena in general and the low-income neurosurgical settings in particular to look into the disparity in the burden of mortality from skull base brain tumor resections. Similarly, identification of the predictors of mortality following skull base brain tumor resection will provide a safer surgical strategy by minimization of preventable risk factors in an attempt to improve the standard of patient care and surgical outcome.

## Methods

A 5-year hospital-based retrospective cohort study was conducted on the incidence and predictors of mortality in patients with skull base tumor resection. The study was conducted from January 2018 to January 2023. The source population of this study was all patients undergoing anterior and middle cranial fossa tumor resection at two of the largest affiliate neurosurgical teaching hospitals of Addis Ababa University, while the study populations were all patients who underwent surgery for anterior and middle cranial fossa tumors fulfilling the inclusion criteria in the study area and period. All patients who had pre-operative, intraoperative, and post-operative clinical and imaging data [computed tomography (CT) scan, magnetic resonance imaging (MRI), or CT angiography] in the electronic patient database registry and image database system of the hospitals were included in the study. Two-dimensional (2D) size measure was applied from pre-operative MRI and CT images. The largest diameter was taken as the size of the tumor among different axis measures. Patients with absent and/incomplete data registry and follow-up imaging were excluded.

### Neurosurgical setting in the study area

In our practice, neurosurgeons with varying experience levels have performed procedures on all types of skull base tumors, regardless of complexity. The far low availability of CT and MRI services in the country also contributed to the delayed diagnosis of tumors with a huge size.

Although surgical adjuncts such as image guidance, neuronavigation, and intraoperative localization of vascular structures (e.g., Doppler, intravascular fluorophores) are important, we have not used these tools to localize major vessels in relation to tumors owing to the unavailability of the gadgets and limited experience of the operating neurosurgeons in our setting. In addition, we do not use adjunctive stereotactic radiosurgery at all due to its unavailability, and the use of conventional radiotherapy is limited due to limited access to it, making surgery the mainstay of treatment in our setting. To reduce the vascularity of the tumor, we have never utilized angiographic (endovascular) embolization due to the absence of endovascular intervention equipment. Furthermore, endovascular interventions like mechanical and chemical angioplasty have never been utilized for patients with inadvertent iatrogenic vascular injury and vasospasm.

Tumor resection has been performed by operating microscopes and microsurgical techniques such as microscissors and bipolar cautery. However, we have never used a Cavitron ultrasonic surgical tissue aspirator (CUSA) due to unavailability. As a result, our procedures have taken longer operating hours, leading to a high incidence of brain contusion, brain edema, and procedure-related vascular insults. In addition, there has never been the utilization of neuromonitoring of any region of the neurosurgical procedure in our setting. The limited access to a dedicated neurocritical intensive care unit and post-operative rehabilitation therapies has contributed to the increased length of hospital stay, morbidity, and mortality in our setup.

### Outcome measure and predictor variables

The outcome variable is Mortality at 3-month post-operative follow-up time (Yes/No). Mortality predictor variables like sociodemographic factors [comorbid factors, smoking, pre-operative Karnofsky performance status (KPS), age, and sex], pre-operative imaging factors (tumor type, location, size, pre-operative radiation/embolization, and vessel encasement), intraoperative surgical factors [surgical approach, extent of resection, intraoperative brain edema, intraoperative brain contusion, vessel manipulation, iatrogenic vessel injury, estimated blood loss (EBL), use of hemostatic agents, and difficulty of securing hemostasis], and post-op factors [clinical course and post-op control imaging factors (extent of resection, tumor bed hematomas, hydrocephalus, pneumocephalus, contusion, brain edema, and cerebral infarction)] were prepared by searching from available scientific literatures.

### Operational definition

Subtotal resection (STR) was defined by the resection of <95% of the tumor.

Near total resection (NTR) was defined by the resection of ≥95% of the tumor.

Gross total resection (GTR) was defined by the resection of 100% of the tumor.

### Data collection tools and procedures

Data collection tool was developed in structured form from previous published literatures by using the Google form and data were collected by the neurosurgeons only to assure quality of data. The data were collected from the patient's electronic record, imaging database system, patient chart, and phone call to patients to get complete data.

### Data quality control

The collected data were checked for completeness, clarity, and accuracy on a daily basis and corrections were made as needed. Data clean up, cross-checking, and double entry were done before analysis. Accurate diagnosis of the pre-operative, intraoperative, and post-operative factors was done by critical evaluation of the peri-operative images and clinical profiles of the patients by the principal investigator and was cross-checked with the neuroradiologists’ comments of the image available on the image database system.

### Data processing and analysis

The coded data on Google form containing demographic, pre-operative imaging factors, post-op clinical factors, and post-op control imaging factors were exported to SPSS version 25. The categorical variables were expressed in frequencies and percentages and compared with *X*^2^ or Fischer’s exact test. Normal distribution of continuous data was checked by histogram and the Shapiro–Wilk test. Mean with standard deviation (SD) was calculated for normally distributed data, while median with interquartile range (IQR) was calculated if the data were skewed.

The incidence rate was calculated for the occurrence of mortality of patients at 3 months after undergoing anterior and middle cranial fossa tumor resection. Univariate analysis was done to see the association of each predictor variable with the mortality of patients. Variables with *p*-value of <0.25 in the univariate analysis were entered to multivariate analysis. The statistical significance was declared at *p*-value <0.05. The measure of association was expressed using odds ratio (OR) and/or adjusted odds ratio (AOR) with 95% confidence interval (CI).

## Result

### Patient demographic characteristics

According to a study done on 266 patients with skull base tumor resection, the median age of the patients with skull base in this study was 37 (±IQR = 17) years, indicating that the average age of the study population was relatively young. In addition, the majority of patients were women, accounting for 65% of the cohort. Furthermore, 15.6% of the patients had comorbidity and more than 95.9% of the patients who underwent skull base tumor resection had a KPS of more than 70%, indicating a relatively good functional status in the majority of these patients (Table 1).

**Table 1:** Demographic characteristics of patients with skull base brain tumor resection in large-volume neurosurgical centers, Ethiopia (*N* = 266).

Item	Variables	Frequency	Percent
Sex	Female	169	63.5
Age	<40	144	54.1
	>40	122	45.9
Comorbidity	Yes	42	15.8
KPS	<70	11	4.1
	≥70	255	95.9
Redo surgery	Yes	29	10.9

### Clinical profile

Headache and loss of vision were the most common clinical presentations among patients with skull base tumors, accounting for 85.7% and 82.7% of cases, respectively. Visual disturbances were common in our study due to the proximity of critical structures involved in vision, such as the optic nerves and chiasm with skull base tumors and relatively delayed presentation at diagnosis. The mean duration of patients’ presenting symptoms was 17.3 (±SD = 11.1) months with minimum and maximum duration of symptoms being 1 and 84 months, respectively. The study included all anterior and middle cranial fossa tumors, with 79.3% arising from the middle cranial fossa and the remaining tumors originating from the anterior cranial fossa. Meningioma was the most common type of tumor, accounting for 68.8% of the cases, with the tuberculum sellae being the most common epicenter of these tumors. The median size of the tumor in this study was 4.9 (±IQR 1.5) cm, indicating that the majority of tumors were of large size (Table 2).

**Table 2:** Tumor characteristics and clinical presentation of patients with skull base brain tumor resection in a large-volume neurosurgical center, Ethiopia (*N* = 266).

Item	Variables	Frequency	Percent
Location (*N* = 266)	Middle cranial fossa	211	79.3
	Anterior cranial fossa	47	17.7
	Both	8	3
Tumors (*N* = 266)	Meningioma	182	68.4
	Pituitary macroadenoma	51	19.2
	Craniopharyngioma	24	9.0
	Other	9	3.38
Epicenter of meningioma (*N* = 182)	En plaque meningioma	2	0.8
	Lateral and/or middle sphenoid wing meningioma (SWM)	29	10.9
	Middle fossa meningioma	1	0.4
	Clinoidal/medial SWM	40	15.0
	Olfactory groove meningioma	31	11.7
	Planum sphenoidale meningioma	17	6.4
	Spheno-orbital meningioma with/without cavernous sinus extension	12	4.5
	TSM	50	18.8
Vessel encasement (*N* = 266)	Grade 0	49	18.4
	Grade 1	81	30.5
	Grade 2	61	22.9
	Grade 3	43	16.2
	Grade 4	19	7.1
	Grade 5	13	4.9
Pre-operative hydrocephalus (*N* = 266)	Yes	25	9.4
Pre-operative edema (*N* = 266)	Yes	123	46.2
Size (*N* = 266) (cm)	<4	91	34.2
	≥4	175	65.8
Midline shift (*N* = 266) (mm)	<5	209	78.6
	≥5	57	21.4
Clinical Presentation (*N* = 266)	Headache	228	85.7
	Weakness	24	9.0
	Seizure	44	16.5
	Sphincter dysfunction	24	9.0
	Vision impairment	219	82.3
	Smell impairment	27	10.2
	Impaired level of consciousness	30	11.2

TSM, Tuberculum Sellae Meningioma.

The most common surgical procedure performed for those tumors was craniotomy, which accounted for 81.6% of the cases while 18.4% of the patients underwent trans-sphenoidal surgery (TSS). More than 60% of the patients underwent GTR or NTR, while 35.7% of the patients achieved subtotal resection. It was found that 22.6% of the 266 patients who had their masses resected had mass effect on follow-up imaging after the procedure, and 6.8% of them had brain swelling during the procedure. The most common post-operative bleeding complications were intracerebral hemorrhage (ICH) at 9.2%, subarachnoid hemorrhage (SAH) at 8.6%, and acute subdural hematoma (ASDH) at 2.6%. Pneumocephalus occurred in 9.8% of the cases and a variable extent of brain contusion occurred in 35.3% (Table 3).

**Table 3:** Intraoperative and post-operative events in patients with skull base brain tumor resection in a large-volume neurosurgical center, Ethiopia (*N* = 266).

Item	Variables	Frequency	Percent
Procedure	Craniotomy	217	81.6
	TSS	49	18.4
Intra-op factors	Brain contusion	83	31.2
	Brain swelling	18	6.8
	Hemostatic agent use	201	75.6
	Difficult hemostasis	37	13.9
EBL (L)	<1	178	66.9
	1–2	73	27.4
	≥2	15	5.6
Extent of resection	NTR or GTR	171	64.3
	STR	95	35.7
Post-op changes on control images	SAH	23	8.6
	ASDH	7	2.6
	ICH	51	19.2
	IVH	25	9.4
	Epidural hematoma (EDH)	5	1.9
	Pneumocephalus	24	9.0
	Mass effect on control CT	60	22.6
Ischemic cerebrovascular complications	Iatrogenic injury	42	15.8
	Vasospasm	11	4.1
Ischemic vascular territories (*N* = 53)	Anterior cerebral artery (ACA)	8	15.09
	Basilar artery	1	1.51
	Major Cortical artery	2	3.77
	Artery of Heubner	2	3.77
	ICA	15	28.30
	Middle cerebral artery (MCA)	12	22.64
	Multiple artery territory	9	16.98
	Perforators	4	7.94
Mortality at 3 months		56	21.1

An ischemic cerebrovascular insult occurred in 19.9% of the patients; of these, 20.7% were related to cerebral vasospasm and 79.3% were iatrogenic vascular injuries. Regretfully, a significant portion of the patients who suffered from vascular insults (79.2%) died at the 3-month post-operative period. It is indeed concerning in that among the patients who experienced mortality after skull base mass resection, a significant proportion had cerebrovascular ischemic insults (Table 3).

Looking into the distribution of mortality across the surgical approaches utilized, craniotomy had a mortality rate of 28.4%, while trans-sphenoidal surgery had 19.5% mortality. Besides this, larger tumors had higher mortality rates; 11.5% for tumor size <4 cm, while 34.6% for tumors of size >6 cm. When we look at the incidence of mortality per the type of tumor histology, 20.8% of operated skull base meningioma and 29.2% of craniopharyngioma cases died (Table 4). However, the statistical analysis showed that neither the particular tumor pathology nor the type of surgical approach used has a statistical association with the mortality at 3 months with a *p*-value of 0.96 and 0.35, respectively. Similarly, achieving near total or gross total resection carried more or less similar mortality rates (20.5% vs. 22.1%, respectively) and had not a significant difference (Table 4).

**Table 4:** The distribution of 3-month operative mortality as a function of some independent variables among patients who underwent skull base brain tumor resection in a large-volume neurosurgical center, Ethiopia (*N* = 266).

Item	Variables	Frequency of mortality	Percent
Procedure	Craniotomy	48	28.4
	TSS	8	19.5
Tumor histology	PMA	10	19.6
	Meningioma	38	20.8
	Glioma	1	33.3
	Craniopharyngioma	7	29.2
Extent of resection	NTR or GTR	35	20.5
	STR	21	22.1
Size classification in centimeters (cm)	Below 4	11	11.5
	4–6	36	25
	Above 6	9	34.6

In a univariate binary logistic regression analysis, several factors including sex, comorbidity, tumor localization, pre-operative edema, tumor size, vascular encasement, iatrogenic vascular insult, vasospasm, brain contusion, intraoperative brain swelling, ICH, SAH, intraventricular hemorrhage (IVH), post-operative ventriculoperitoneal shunt (VPS), pulmonary embolism (PE), hospital- associated infection (HAI), and extubation time were considered for multiple variable logistic regression to predict mortality at 3 months following skull base tumor resection. Overall, hospital-acquired infection, interop vascular accident, delayed extubation, and brain contusion were the major complications faced in our series (Table 5).

**Table 5:** A 3-month operative mortality predictors in patients with skull base brain tumor resection in a large-volume neurosurgical center, Ethiopia (*N* = 266).

Item	Variables	Mortality at 3 months		COR (95% CI)	*P*-value	AOR (95% CI)	*p*-value
		Dead	Alive				
Sex	Female	40	129	1.57 (0.83–2.99)	0.17	1.53 (0.47–5.04)	0.47
	Male	16	81	1		1	
Comorbidity	Yes	6	36	1.72 (0.69–4.33)	0.246	1.69 (0.36–7.80)	0.50
	No	50	174	1		1	
Location	Middle and both	50	169	2.02 (0.81–5.03)	0.131	0.93 (0.17–5.06)	0.94
	Anterior	6	41	1		1	
Pre-op edema	Yes	30	93	1.45 (0.83–2.62)	0.217	3.19 (0.82–12.43)	0.09
	No	26	117	1		1	
Size of tumor (cm)	Below 4	9	82	1		1	
	4	47	128	3.35 (1.56–7.19)	0.002*	1.59 (0.30–8.24)	0.58
Vascular encasement	Grade 0–2	12	118	1		1	
	Grade 3–5	44	92	4.70 (2.34–9.41)	0.000*	1.260 (0.34–4.67)	0.73
Iatrogenic vascular injury	Yes	32	10	26.66 (11.66–60.95)	0.000*	28.76 (6.12–135.08)	0.000*
	No	24	200	1		1	
Vasospasm	Yes	6	5	4.92 (1.44–16.77)	0.011*	8.176 (0.96–69.36)	0.054
	No	50	205	1		1	
Brain contusion	No	29	154	1		1	
	Yes	27	56	2.56 (1.39–4.70)	0.002*	4.01 (0.83–19.34)	0.084
Intraoperative brain swelling	Yes	10	8	5.49 (2.05–14.67)	0.062	6.57 (1.03–41.93)	0.047
	No	46	202	1		1	
ICH	No	34	181	1		1	
	Yes	22	29	4.04 (2.08–7.85)	0.000*	1.94 (0.44–8.57)	0.382
SAH	No	39	204	1		1	
	Yes	17	6	14.82 (5.50–39.96)	0.000*	2.63 (0.50–13.96)	0.26
IVH	No	38	203	1		1	
	Yes	18	7	13.74 (5.37–35.14)	0.000*	6.32 (1.19–33.63)	0.031*
Post-op VPS	No	53	206	1		1	
	Yes	3	4	2.95 (0.63–13.42	0.170	6.831 (0.49–95.81)	0.154
PE	No	51	207	1		1	
	Yes	5	3	6.77 (1.56–29.24)	0.010*	6.19 (0.64–59.76)	0.115
HAI	No	29	186	1		1	
	Yes	27	24	7.22 (3.67–14.17)	0.000*	6.96 (2.04–23.67)	0.002*
Extubation time (h)	<24	19	191	1		1	
	≥24	45	11	41.12 (18.29–92.49)	0.000*	12.89 (4.19–40.34)	0.000*

COR, crude odds ratio.

* shows the statistically significant findings only.

Prior to adjustment for risk factors, the following were associated with statistical significance with 3-month mortality after skull base tumor resection: size larger than 4 cm (OR = 3.35, 95% CI: 1.56–7.19, *p* = 0.002), vascular encasement grade 3–5 (OR = 4.70, 95% CI: 2.34–9.41, *p* = 0.000), iatrogenic vascular injury (OR = 26.66, 95% CI: 11.66–60.95, *p* = 0.000), vasospasm (OR = 4.92, 95% CI: 1.44–16.77, *p* = 0.011), brain contusion (OR = 2.56, 95% CI: 1.39–4.70, *p* = 0.002), ICH (OR = 4.04, 95% CI: 2.08–7.85, *p* = 0.000), SAH (OR = 14.82, 95% CI: 5.50–39.96, *p* = 0.000), IVH (OR = 13.74, 95% CI: 5.37–35.14, *p* = 0.000), PE (OR = 6.77, 95% CI: 1.56–29.24, *p* = 0.010), HAI (OR = 7.22, 95% CI: 3.67–14.17, *p* = 0.000), and extubation time exceeding 24 h (OR = 41.12, 95% CI: 18.29–92.49, *p* = 0.000) (Table 5).

Nevertheless, in subsequent multivariable logistic binary regression analysis, intraoperative iatrogenic vascular insult (AOR = 28.76, 95% CI: 6.12–135.08, *p* = 0.000), IVH (AOR = 6.32, 95% CI: 1.19–33.63, *p* = 0.031), HAI (AOR = 6.96, 95% CI: 2.04–23.67, *p* = 0.002), and extubation time exceeding 24 h (AOR = 12.89, 95% CI: 4.89–40.34, *p* = 0.000) were found to be significantly associated with mortality at 3 months following skull base tumor resection (Table 5).

## Discussion

To the authors’ best knowledge, predictors of mortality among patients who underwent surgical resection of skull base tumors have never been studied even if the practice of skull base surgery started in 2010 in Ethiopia, a low-income setting. Similarly, neurosurgical resection to a variety of complex and difficult to access skull base tumors continues being the definitive and gold standard treatment option available to date. This surgical treatment has been improving technically and has become safer especially in recent times because of the refinements of the surgical techniques and the continuously improving surgical equipment. This however is sometimes associated with dreadful complications that lead to grave morbidity and mortality; especially in low-income settings, where both the distribution and safety of neurosurgical services are neither sufficient nor equally safe as is in the high-income neurosurgical settings.

Accordingly, this study showed that there were 21.1% mortalities following skull base tumor resections. This operative mortality was much higher than the operative mortality [10% (*N* = 100)] for skull base meningioma reported in the same institution in 2019 (8) and is slightly higher compared with other developing neurosurgical centers like Nigeria with a mortality rate of 19.6% (*N* = 51) according to a study conducted in 2012 (9). Similarly, the overall mortality rate of our center was much higher than that of the Cuban neurosurgical center, where 16% (*N* = 55) of the patients died after undergoing skull base tumor resection in 2022 (10). By contrast, the mortality rate of skull base tumor surgery in better-income centers like Brazil have persistently been about 5% (*N* = 49) even for complex and giant skull base tumors (11). Overall, the mortality rate in our setting is one of the highest in the world, which partly implicates the unfair distribution and access to quality neurosurgical services. In other words, this is an indicator of the continued disparity of quality access to the global neurosurgical services.

When the immediate causes of mortality were looked for, it appeared that our setup had higher incidences of ischemic cerebrovascular complications as nearly half of the mortalities (42.9%) were purely ascribed to surgery-related vascular insults, which include iatrogenic vascular injury and cerebral vasospasm. Only 41.1% of mortalities were from non-ischemic cerebrovascular-related causes, while the remaining 16% deaths were related to both ischemic cerebrovascular and non-ischemic cerebrovascular complications. This study pointed out that, while surgery-related vascular insults needs special due attention, the non-ischemic cerebrovascular causes of mortalities still played nearly an equal role in the causation of mortality.

Given that there was a high rate of 3-month mortality in patients with skull base tumor resection, we systematically looked into pre-operative tumor and patient characteristics, intraoperative events during the surgery, and post-operative factors that contributed to the mortalities. The factors that showed significant association with mortality are discussed separately.

### Pre-operative tumor and patient factors

Before adjusting for cofounders, tumor size >4 cm was likely to have three times increased mortality risk than smaller tumors (*p* < 0.002). Similarly, a higher grade vascular encasement (grade 3–5) was found to have more than four times increased mortality risk (*p* < 0.000). The presence of statistical association between mortality and higher grade encasement and larger size tumors sounds scientific because a larger-sized tumor tends to have higher grade vascular encasement that maximizes the potential for cerebrovascular and non-cerebrovascular complications such as contusion, brain swelling, and increased blood loss during surgery. By the same token, the occurrence of either cerebrovascular or non-vascular insults increased the mortality risk. This is consistent with other studies that tried to look for the association between the size and vessel encasement with the occurrence of surgery-related ischemic cerebrovascular complications and functional outcomes as defined by the modified Rankin scale (MRS) (12). However, after adjusting for confounders, neither size > 4 cm [AOR = 1.59 (0.30–8.24), *p* < 0.58] nor higher grade of vascular encasement [AOR = 1.260 (0.34–4.67), *p* < 0.73] was shown to have statistically significant association with mortality in our study. However, this still is clinically significant and cautious planning is vital for patients with larger-sized tumors with or without vascular encasement.

Notably, the mean duration of patients’ presenting symptoms was 17.3 (±SD = 11.1) months and is much delayed compared with the median of 24 days with minimum and maximum duration of symptoms at the time of diagnosis being 7 and 65 days, respectively, according to a study that was conducted in the UK. This degree of discrepancy might be related with the poor access to diagnostic neuroimaging services, inadequate knowledge to consider the diagnosis of brain tumors by primary care practitioners, and poor health seeking behavior of patients in our setting (13).

### Intraoperative factors

Before adjusting for other variables, intraoperative brain contusion increased the odds of mortality more than two times compared with those patients with no brain contusion (*p* < 0.002). There is paucity of evidence regarding the absolute risk of mortality posed by cerebral contusion after skull base tumor resection, but the mechanism by which increased mortality ensued can be explained by the cytotoxic and vasogenic brain edema, which in turn increases intracranial pressure and altered regulation of cerebral blood flow and cerebral perfusion pressure; ultimately leading to cerebral metabolism alteration, permanent damage to cerebral tissue, and death (14). Normally, contusion is thought to occur as a result of the ongoing bleeding of microvessels due to the underlying traumatic episode and it undergoes progression at different post-operative times; the peak times being the first 24 h, 3–5 days, and 7–9 days after the inciting event. The nuclear factor named kappaB, which leads to apoptosis of the contused neuronal cells, and specificity factor 1, which causes fragmentation of the vessels, facilitate the contusion progression, thereby increasing the mortality risk of patients (14–16).

Similarly, it appeared that the presence of both intracerebral and subarachnoid hemorrhage had a statistically significant odds of 4 and 14 times increased mortality risk, respectively (*p* < 0.000) (17). The possible pathophysiological mechanisms that contribute to mortality include the massive neuronal loss related with direct neurotoxin effect (e.g., hemoglobin, heme, thrombin, iron, and complement-induced injury) of primary hemorrhage on neural structures, cerebral infarction from subarachnoid hemorrhage induced vasospasm, refractory cerebral edema leading to brain stem herniation, hydrocephalus, and medical complications (e.g., fatal arrhythmia) (17–19).

After adjusting for confounders, our study clearly showed that the presence of intraoperative iatrogenic injury showed a statistically significant association with mortality from skull base tumor resection and carries about 28 times increased risk of mortality than in those having no vascular injury (*p* < 0.000). The lethality rate of vascular injury in our series was 79.3%, which is far higher than that reported (10%) even after internal carotid artery (ICA) injury (20). While the explanation for mortality related with iatrogenic vascular injury is straightforward, it includes ischemia-related malignant brain swelling, massive blood loss during vascular injury that compromises cerebral perfusion, excitotoxicity from glutamate and oxidative stress-induced intrinsic and extrinsic apoptosis, ferroptosis, parthanatos, pyroptosis, necroptosis, and autophagic cell death, just to mention few among many plausible explanations (21). The association between iatrogenic vascular injury and mortality is consistent with the study that was conducted to assess internal carotid artery injury during endonasal endoscopic surgeries (22). Accordingly, 7.14% (2/28) of the patients who developed iatrogenic injury had died (22). However, the mortality rate following iatrogenic vascular injury was quite high 79.2% (42/53) compared with the two mortalities from 28 ICA injuries. This was further strengthened by the full recovery of 19 patients and one death out of the 20 patients with iatrogenic internal carotid arteries during an endonasal endoscopic resection of skull base tumors with reported ICA patency preservation rate of 83.3% following the advent of stent graft in 2016 (23). This huge difference in mortality once vascular injury occurred could be due to the absence of Angio suites for endovascular procedures in our setting, which the authors underscore to change so that the quality of global neurosurgery improves fairly.

Similarly, our study showed that an intraoperative brain swelling had more than six times adjusted odds of mortality in patients who underwent skull base tumor resections [AOR = 6.57 (1.03–41.93), *p-*value = 0.047]. Although there is no ample and methodologically sound clinical study that assesses the occurrence and outcomes of intraoperative brain swelling during brain tumor resection in general and skull base tumor resections in particular, many case reports and series showed that it poses a significant increased risk of morbidity and mortality (24). Multiple causes like ischemia, hypoxia, and overzealous manipulation of brain, hemodynamic failure, and cerebral venous outflow obstruction have been entertained during the intraoperative procedure (24).

Furthermore, our study pointed out that IVH carried an adjusted increased mortality risk of six times compared with in those having no IVH (*p*-value < 0.031). The contribution of higher mortality related with IVH in patients who underwent skull base tumor resection could be due to the direct toxic effects of the blood onto the sensitive brain parts and partly because these patients tend to develop hydrocephalus, which in turn leads to the placement of external ventricular drain (EVD) and EVD-associated ventriculitis, thereby contributing additional risk of mortality from the direct toxic effect of blood, hydrocephalus, and ventriculitis. Even if there is a paucity of evidence that was determined to dedicatedly assess the mortality impact of IVH in patients who underwent skull base resection, a pooled analysis that was conducted to assess the mortality impact of IVH in patients with ICH showed to increase death rate to 51.2% compared with 19.5% in cases where ICH alone without IVH occurs (25).

### Post-operative factors

Due to the intricacy of neurosurgical management of skull base tumors, a highly specialized post-operative neurointensive care unit is equally important as are the proper pre-operative planning and meticulous intraoperative surgical techniques in dealing with the surgical removal of various types of skull base tumors (26). Otherwise, the mortality of these patients will exponentially increase.

Accordingly, patients with hospital-acquired infections, severe cranial trauma [ventriculitis, cerebrospinal fluid (CSF) leak, and meningitis], and ventilator-associated pneumonia (VAP) had about seven times adjusted odds of mortality after adjusting for confounders (*p* < 0.002). This increased mortality is obvious in that sepsis and its worst form, septic shock and multiple end-organ failure, in itself are independent risk factors for mortality. The issue however is the unacceptably high incidence of post-operative infection in our setup (19.2%). This high infection rate could be an indicator of a poor quality outcome measure per global neurosurgery standards. The absence of a well-dedicated, equipped neurointensive care unit that is staffed by a well-trained team of health personnel, well-equipped operating room, and some technical factors during surgery partly contribute to this high incidence of infection; which ultimately snatches off patients’ lives. This warrants a notable mention in that infection prevention in low-income settings is and should be one of the top priority areas of quality improvement.

Similarly, delayed extubation time (more than 24 h) was shown to have statistically significant association with increased mortality in patients with skull base tumor resection (*p*-value < 0.000). This is in agreement with the existing scientific evidence even if high quality data are lacking with regard to the decision to extubate patients. The association between delayed extubation and mortality could probably be explained by the increased risk of ventilator-associated pneumonia without decreasing the risk of extubation failure (27, 28).

In addition, pulmonary embolism [OR = 6.77(1. 56–29.24)] had statistically significant association with mortality (*p* < 0.01) and it occurred in 3% of the patients with a fatality rate of 62.5% (*N* = 8). This is supported by a study that was conducted to assess the global incidence and case fatality of pulmonary embolism following major surgery. Accordingly, the incidence was high (0.3%–30%) with high mortality rate (16.9%–31%) despite a widespread use of thromboembolic preventive measures (29).

In summary, this study came to an observation that patients who had ischemic cerebrovascular insults (iatrogenic injury or vasospasm) tended to have other non-ischemic complications (HAI, PE, and delayed extubation) as the occurrence of the former could lay a fertile ground for the latter, especially in setups where there is no dedicated neurointensive care unit. Hence, thorough pre-operative planning and meticulous intraoperative execution of the surgery are of paramount importance in minimizing post-operative complications, morbidity, and mortality.

### Strengths and limitations

The study's strength lay in the fairly large study population in a low-income setting and the comprehensive involvement of a variety of skull base tumors. The continued effort of neurosurgeons to improve neurosurgical service in low-income settings, even in the absence of necessary neurosurgical gadgets and equipment, is also commendable.

However, the main limitation of the study was its retrospective nature. In addition, the absence of adjuvant treatment using both stereotactic radiosurgery and conventional radiotherapy, CUSA, endovascular treatment options, and a dedicated neurointensive care unit in our setup contributed to a higher incidence of vascular insults and surgery-related mortality of patients undergoing surgery for complex skull base tumors. Furthermore, the routine post-operative follow-up imaging for pituitary tumors was not obtained peri-operatively while the patients were admitted, and only patients with challenging pituitary tumors had follow-up imaging in our setup. This understandably increased the mortality rates for pituitary tumors. The limited availability of CT and MRI services in the country also contributed to the delayed diagnosis of the tumor.

Despite these limitations, the progress in skull base tumor surgery practice in Ethiopia is promising and has helped numerous patients who otherwise had no other options. Because the tumors are huge and very challenging even for the best centers, the way forward for skull base tumor surgery practice in Ethiopia is optimistic.

## Conclusion

Mortality from skull base tumor resection remains high in our setting. Holistic pre-operative surgical planning, meticulous intraoperative execution of procedures, and post-operative dedicated follow-up of patients alongside quality improvement works on identified risks is strongly recommended to improve patients’ outcomes. The urgent need for setup improvement and further training of neurosurgeons is also underscored.

## Data Availability

The raw data supporting the conclusions of this article will be made available by the authors, without undue reservation.
